# Acute Effects of Nicotine on Non-Drug-Related Reward in Smokers and Non-Smokers

**DOI:** 10.1093/ntr/ntae278

**Published:** 2025-02-05

**Authors:** Nicola Rycroft, Catherine Kimber, Emke S E Brazier, Lynne Dawkins

**Affiliations:** Nicotine, Tobacco and Vaping Research Group, Division of Psychology, London South Bank University, 103 Borough Road, London SE1 0AA, UK; Nicotine, Tobacco and Vaping Research Group, Division of Psychology, London South Bank University, 103 Borough Road, London SE1 0AA, UK; Nicotine, Tobacco and Vaping Research Group, Division of Psychology, London South Bank University, 103 Borough Road, London SE1 0AA, UK; Nicotine, Tobacco and Vaping Research Group, Division of Psychology, London South Bank University, 103 Borough Road, London SE1 0AA, UK

## Abstract

**Introduction:**

Nicotine increases the reward value of non-drug-related stimuli in animals and dependent smokers; however, research on people who are not dependent on nicotine is limited. This study aimed to explore whether nicotine delivered by oral spray can enhance responding to self-selected sensory rewards in both smokers and non-smokers.

**Aims and Methods:**

Minimally abstinent smokers (*n* = 30) and non-smokers (*n* = 31) completed subjective ratings of nicotine withdrawal, and received either 2 mg nicotine or placebo oral spray and visual analogue scales to measure the perceived effects of the spray. An operant conditioning task (Applepicker) that required button clicks to find apples was completed twice, with and without a reward of 30 seconds of pre-prepared music for each reinforcer earned. Measures taken were the number of apples found (reinforcers), number of clicks (responses), and time spent on the task (in seconds).

**Results:**

There were no differences between smokers and non-smokers on ratings of nicotine withdrawal or effects of the spray. All participants spent longer searching for apples, earned more reinforcers, and produced more responses when listening to music. Nicotine administration led to a higher number of reinforcers earned and, when music was playing, an increase in a number of responses. A three-way interaction revealed that non-smokers who had received nicotine spent the longest searching for apples.

**Conclusions:**

Nicotinic enhancement of sensory rewards was seen in non-smokers only which cannot be accounted for by learned associations with nicotine or reversal of withdrawal effects. Smokers, however, may require higher doses of nicotine to achieve the same effect.

**Implications:**

Nicotinic enhancement of sensory rewards was demonstrated in non-smokers, but not in everyday smokers, suggesting it is unlikely to be related to nicotine dependence or learned associations between nicotine and pleasure. The absence of this effect in smokers suggests that higher levels of nicotine than those obtained from 2 mg oral sprays may be required to achieve enhancement of reward in people who regularly consume nicotine. For nicotine replacement (including e-cigarettes) to become more effective at reducing anhedonia during quit attempts, smokers may require nicotine doses that more closely replicate levels achieved through smoking.

## Introduction

Nicotine is the main psychoactive ingredient of tobacco smoke and is thought to underlie many of the subjective and rewarding effects of smoking cigarettes.^1^ Subjective effects of nicotine are, however, quite subtle, especially when compared to other addictive drugs such as alcohol and heroin. Despite these relatively weak effects, success rates for stopping smoking have remained relatively modest at just under 27% in the United Kingdom.^2^ It seems likely, therefore, that nicotine delivered via tobacco smoke provides smokers with some benefits that make long-term abstinence hard to achieve. Previous studies have demonstrated that nicotine can increase the reward value of a range of non-drug-related stimuli, such as music or videos.^3,4^ The aim of this study is to explore whether nicotine can enhance responding for rewards using an alternative (ie not smoked) form of nicotine delivery in both smokers and non-smokers.

Nicotinic enhancement of reward has been robustly demonstrated in animals. For example, Chaudhri et al.^5^ found rats given nicotine that was not contingent upon their own behavior increased operant responding for a mildly reinforcing visual and auditory stimulus. Palmatier et al.^6^ found nicotine led to faster learning of how to respond for rewards and increased perseverance to obtain the rewards. These effects of nicotine appeared to be greater for auditory/visual stimuli than for a 4% sucrose solution;^7^ however, nicotine did enhance responding for a 26% sucrose solution.^8^ Nicotinic enhancement of reward has become so well established in rats, it has been used as a paradigm to test the role of dopamine receptor subtypes and opioid receptors in the pharmacology of the brain’s reward system.^8,9^ In animals, nicotinic enhancement of reward appears to be due to stimulation of the α4β2 nicotine receptor in the ventral tegmental area,^10^ the same subtype most closely linked with the addictive properties of nicotine.^11^

In humans, nicotine can enhance responding for small monetary rewards in both smokers^12^ and non-smokers.^13^ Smokers rated faces as more attractive after smoking a nicotinized compared to a denicotinized cigarette^14^ and smoking enhanced self-reported pleasure obtained from viewing movie clips.^15^ Using a conditioned place preference paradigm (CPP) adapted for use in humans, Palmisano et al.^16^ found a 4 mg nicotine lozenge-induced CPP only in occasional smokers with a score on the Fagerstrom Test of Nicotine Dependence of 1 or higher. However, the reward in this study was chocolates, and nicotine’s action as an appetite suppressant, may have reduced desire for chocolate whilst completing the task. In non-smoking humans, 2 mg nicotine delivered by lozenge may also increase activation of the nucleus accumbens when monetary rewards are anticipated.^17^ These studies do imply that nicotine may act to increase the enjoyment or reward value of non-drug related stimuli but many of the effects were seen in smokers. It is unclear, therefore, whether these findings can be explained via pharmacological effects of nicotine or if sensory, behavioral, or conditioned responses to smoking may be involved.

The most direct tests of nicotinic enhancement of reward come from studies that have used operant responding for rewards, similar to the methods used in animals. Using the Applepicker task, where displays of trees have to be clicked upon (responses) to find apples (reinforcers), smokers who had recently smoked a cigarette that contained nicotine increased operant responding for music,^3^ money,^18^ and video rewards^19^ as compared to smokers who had smoked a denicotinized cigarette or who had abstained from smoking. These effects may be larger in women than in men.^4^ Using an e-cigarette containing a nicotine concentration of 36 mg/ml, nicotine enhanced responding for video rewards in overnight abstinent smokers.^20^ Nicotine delivered via patch (14 mg) and nasal spray (2 mg) had similar effects in overnight abstinent smokers.^21^ These latter studies suggest that this effect of nicotine is not, therefore, related simply to the experience of smoking a cigarette and the sensory aspects of smoking.

Very few studies have, however, explored this effect of nicotine in people who are not dependent upon nicotine. Kirshenbaum and Hughes reported nicotine delivered by e-cigarette to occasional (less than once a month) users of nicotine products increased the reward value of playing video games^22^ and Perkins and Karelitz found nicotinic enhancement of reward occurred equally in both dependent and non-dependent smokers.^3^ When testing smokers, attempts can be made to account for the reversal of withdrawal as an explanation for beneficial effects of nicotine. It is, however, harder to remove the influence that learned associations between nicotine and pleasure may have on any beneficial effects. This study, therefore, recruited both daily smokers and non-smokers in order to explore the effects of 2 mg nicotine oral spray on the enhancement of reward in both groups. As audio-visual rewards appear to show the most robust enhancement effects,^21^ participants in this study were asked to create their own playlists of music to listen to as the reward. The aim was to explore whether nicotine can enhance responding for sensory rewards in non-smokers and whether this effect can be demonstrated in smokers via a novel form of nicotine administration.

## Methods

### Participants

Participants were recruited from the student body at London South Bank University. Smokers (*n* = 30) were mostly female (*n* = 27) with a mean age of 25.16 (SD 5.29) years. They all smoked at least five cigarettes per day, had smoked every day for a minimum of 6 months, and had a mean score of 1.93 (SD 2.54) on the Fagerström Test of Cigarette Dependence (Heatherton et al.^23^). Smokers were asked to “smoke as usual” on the day of the experiment but to abstain for 2 hours immediately before their session in order to avoid reversal of withdrawal effects during the study. Non-smokers (*n* = 31) were also mostly female (*n* = 23) with a mean age of 23.58 (SD 6.85) years. Non-smokers were defined as having never smoked cigarettes on a daily basis and had smoked fewer than 100 cigarettes in their lifetime.

### Measures and Materials

#### Fagerstrom Test of Cigarette Dependence

The Fagerstrom Test of Cigarette Dependence (FTCD) was used to measure the intensity of the participant’s physical nicotine addiction.^23,24^ It is a six-item self-report scale, and total scores range from 0 (low cigarette dependence) to 10 (high cigarette dependence).

#### Mood and Physical Symptoms Scale

A revised version of the Mood and Physical Symptoms Scale (MPSS) was used to measure the extent of six common nicotine withdrawal symptoms (depressed, irritable, anxious, restless, hungry, unable to concentrate) that participants may have been experiencing at the moment of completing the scale.^25^ Responses were recorded using a five-point scale (Not at all = 0, Slightly = 1, Somewhat = 2, Very = 3, Extremely = 4). Total scores can range from 0 to 20, with higher scores representing an increased experience of withdrawal symptoms. The scale had good reliability, *α* = 0.830 from the measure taken at the start of the experimental session. Cravings were measured by a single item, “How strong is your desire to smoke right now,” answered on a 7-point scale with 1 = not at all strong and 7 = extremely strong.

#### Positive/Negative Subjective Effects of Nicotine

Participants were asked to complete visual analog scales to assess their subjective response to the oral sprays. Each item was rated from 0 (“not at all”) to 100 (“extremely”) by placing a cross on a line. Nine items related to positive effects of the spray, for example, “the mouth spray has made me feel more awake” and “the mouth spray is pleasant.”^26^ Reliability between individual positive items from the start of the experimental session was moderate, *α* = 0.534. Twenty-one items related to negative effects of the spray, for example “nausea/feeling sick,” “mouth irritation,” and “salivation.”^27^ Reliability between individual negative items from the start of the experimental session was good, *α* = 0.902. The mean of responses to all nine positive and all 21 negative items was calculated to create scores that ranged from 0 to 100 for both positive and negative subjective effects.

#### Nicotine/Placebo Spray

Nicotine, 2 mg, was delivered by oral spray (Nicorette QuickMist 1 mg spray, McNeil Products Ltd, Maidenhead, Berkshire), freshmint flavor. The placebo spray was breath freshener mint flavored mouth spray. Both the active and placebo sprays were concealed using black electronic tape and only one type of spray was visible to each participant. The study was single-blind, as the experimenter was able to tell which spray was active or placebo. A 2 mg dose was selected for this study to reduce the likelihood of negative effects, such as dizziness and nausea, in non-smokers.

#### Applepicker Task

The Applepicker Task was programmed using e-prime 3.0 and based on the description given by Perkins et al.^3,18^ Participants viewed a screen that contained between 4 and 12 trees. Each tree had to be clicked (the response) in order to find an apple (the reinforcer). A cross appeared over trees that had been clicked to remove any working memory load from the task. After each tree was crossed out or if an apple was found the participant was presented with another display of trees. An “I quit” button was displayed at the bottom of every screen and participants were reminded that they could stop searching for apples at any time. The first apple was found after 10 trees had been clicked. Subsequent apples were found on a progressive ratio schedule, with the number of responses required increasing by 50% after each apple was found (PR50). The task was programmed to allow finding a maximum of 15 apples and all participants quit before this number was reached. Each apple found could “earn” 30 seconds of the participant’s pre-prepared playlist of music. Music was controlled by the experimenter using the participant’s own smartphone. Music started when the first apple was found and continued to play until the participant chose to quit or the accumulated reward time elapsed. Participants listened to music via their own smartphones and headphones. Three measures were taken from the Applepicker task, total number of apples found (reinforcers), total number of trees clicked (responses) and time spent on task before clicking the “I quit” button.

### Procedure

Participants were fully briefed and contraindications for nicotine use were explained prior to the experimental session. Participants attended one session at the laboratory. After providing written informed consent all participants were asked to provide a measure of exhaled carbon monoxide via Bedfont Pico Smokerlyser to encourage compliance with the request not to smoke for 2 hours. Smokers were asked to complete the FTCD and all participants completed the MPSS and a practice version (maximum of three apples found) of the Applepicker task. Two squirts from the oral spray (either nicotine or placebo) were administered by the experimenter. During the 2–3 minutes for the initial effects of the spray to be felt, participants’ pre-prepared music playlist was checked by the experimenter. Participants were then asked to complete the positive/negative effects of nicotine scales for the first time before the full version of the Applepicker task. This allowed for the ApplePicker task to commence whilst nicotine levels were likely to be rising, or close to maximum. Nicotine levels rise quickly after oral spray administration, reaching 5 ng/ml within 2 minutes^28^ and *t*_max_ varying between 5 and 10 minutes.^28,29^ The Applepicker task was completed twice either with reward (30 seconds of music for each apple found) or without reward (no music played). Task order was counterbalanced between participants and a 2 (task order: music first or second) x 2 (Condition: Reward vs no reward) Analysis of variance (ANOVA) confirmed there were no main effects or interactions with task order for number of apples found, total number of trees clicked, and time spent on task (*p*’s > 0.143). After quitting the Applepicker task, participants completed the MPSS and positive/negative effects of nicotine scales again.

### Data Analysis

Data were analyzed using SPSS v.27. Independent group *t*-tests were used to identify differences between smokers and non-smokers in nicotine withdrawal symptoms, craving for nicotine, and breath-expired CO at the start of the session. Supplementary analysis to look for changes during the testing session in these measures is available here (Tables S1 and S2). Subjective responses to the nicotine sprays, both positive and negative, were analyzed using a 2 (time: time 1 vs. time 2) × 2 (smoking status: smoker vs. non-smoker) × 2 (spray type: nicotine vs. placebo) mixed ANOVA. “Time 1” refers to data collected within 1–2 minutes of administering the spray. “Time 2” refers to data collected post-completion of the Applepicker task. Performance on the Applepicker task was analyzed using a 2 (Condition: Reward vs. no reward) × 2 (smoking status: smoker vs. non-smoker) × 2 (spray type: nicotine vs. placebo) mixed ANOVA.

## Results

### Mood and Physical Symptoms Scale, Craving and Breath-Expired CO

At the start of the experimental session, smokers showed higher levels of craving for nicotine but were not experiencing higher levels of withdrawal symptoms. Higher levels of breath-expired CO amongst smokers confirmed smoking status (see Table 1).

**Table 1.** Mean (SD) and t-test results for the MPSS, craving, and breath-expired CO

	Smokers	Non-smokers	*t* (df = 59)	*p*
CO (ppm)^a^	7.86 (4.45)	2.00 (0.82)	7.099	<0.001
Craving^b^	2.80 (1.50)	1.12 (0.43)	5.894	<0.001
Depressed	0.96 (0.61)	1.10 (0.94)	–0.636	0.527
Irritable	1.20 (0.85)	1.16 (1.03)	0.159	0.874
Anxious	1.20 (0.76)	1.52 (1.12)	–1.284	0.204
Restless	1.33 (0.80)	1.49 (1.21)	–0.572	0.570
Hungry	1.57 (0.97)	1.67 (1.07)	–0.421	0.675
Unable to concentrate	1.37 (0.85)	1.16 (1.10)	0.815	0.419
MPSS Total^c^	7.63 (2.89)	8.10 (5.24)	–0.430	0.669

^a^Levene’s test significant, df = 30.88.

^b^Levene’s test significant, df = 33.57.

^c^Levene’s test significant, df = 47.08.

### Positive/Negative Subjective Effects of Nicotine

Main effects of time revealed that subjective responses to the sprays were higher at time 1, within a minute or two of administration, than at time 2, approximately 5–6 minutes later for both positive and negative effects (see Table 2). A main effect of spray type for negative effects (*F*_(1,57)_ = 8.946, *p* = .004, η_p_^2^ = 0.136) showed that the nicotine spray was perceived as more unpleasant (mean = 23.38, SE = 2.46) than the placebo spray (mean = 12.72, SE = 2.58) at both timepoints. All other main effects and interactions from the visual analog scales were not significant (*p*’s > 0.148) suggesting that both sprays were tolerated equally well by smokers and non-smokers.

**Table 2.** Mean (S.E.), *F*, *p*, and η_p_^2^ values from the main effect of time on the positive and negative subjective effects of nicotine visual analog scales

	Time 1	Time 2	*F* (1,57)	*p*	Ƞ_p_^2^
Positive	27.54 (1.72)	23.54 (1.72)	8.067	0.006	0.797
Negative	23.18 (2.45)	12.92 (2.59)	12.344	0.001	0.932

### Applepicker

#### Number of Apples Found

More apples were found under the reward condition (M = 7.10, SE = 0.19) than the non-reward condition (M = 5.42, SE = 0.21), *F*_(1,57)_ = 52.599, *p* < .001, η_p_^2^ = 0.480. More apples were found after administration of nicotine (M = 6.65, SE = 0.22) than placebo (M = 5.84, SE = 0.24), *F*_(1,57)_ = 6.229, *p* = .015, η_p_^2^ = 0.099. There was no main effect of smoking status and no interactions between condition, smoking status, and spray type (*p*’s > 0.154).

#### Number of Trees Clicked

**Figure 1.**
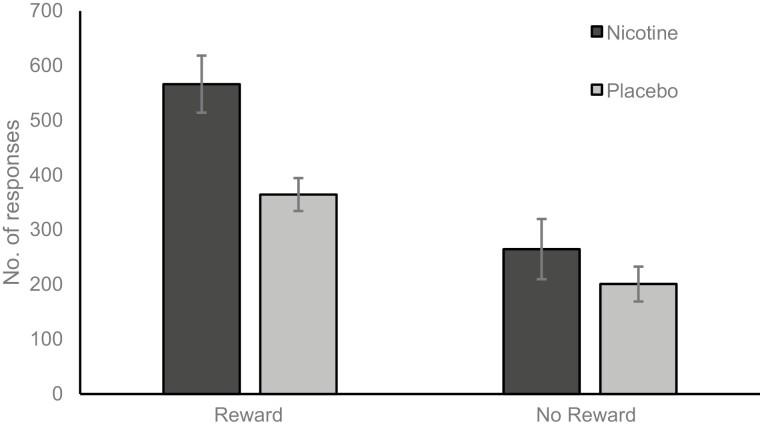
Number of responses made after nicotine or placebo with and without music rewards.

**Figure 2.**
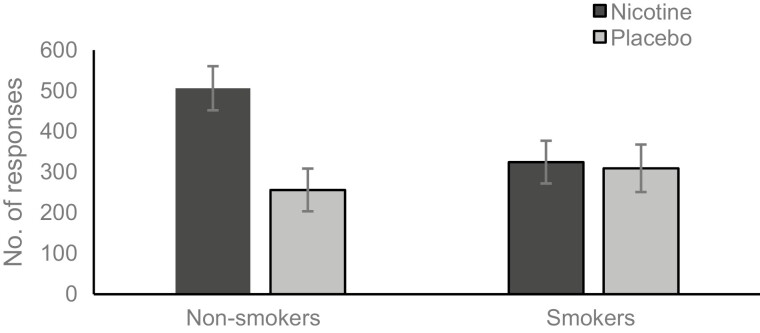
Number responses made after nicotine or placebo by smokers and non-smokers.

Number of clicks was higher under the reward condition (M = 465.27, SE = 37.97) than the non-reward condition (M = 232.82, SE = 21.90), *F*_(1,57)_ = 59.571, *p* < .001, η_p_^2^ = 0.511. More clicks were made after administration of nicotine (M = 415.37, SE = 37.30) than placebo (M = 282.72, SE = 39.31), *F*_(1,57)_ = 5.992, *p* = .017, η_p_^2^ = 0.095. There was no main effect of smoking status (*p* = .241). The significant main effects were qualified by a two-way interaction between condition and spray type, *F*_(1,57)_ = 5.241, *p* = .026, η_p_^2^ = 0.084. More clicks were made after the administration of nicotine when music rewards were earned (see Figure 1). There was also a two-way interaction between smoking status and spray type, *F*_(1,57)_ = 4.704, *p* = .034, η_p_^2^ = 0.076, showing a higher number of clicks amongst non-smokers given nicotine compared to non-smokers given placebo or smokers given either spray (see Figure 2). The three-way interaction between condition, smoking status, and spray type did not reach significance (*p* = .058).

#### Time Spent on Task

**Figure 3.**
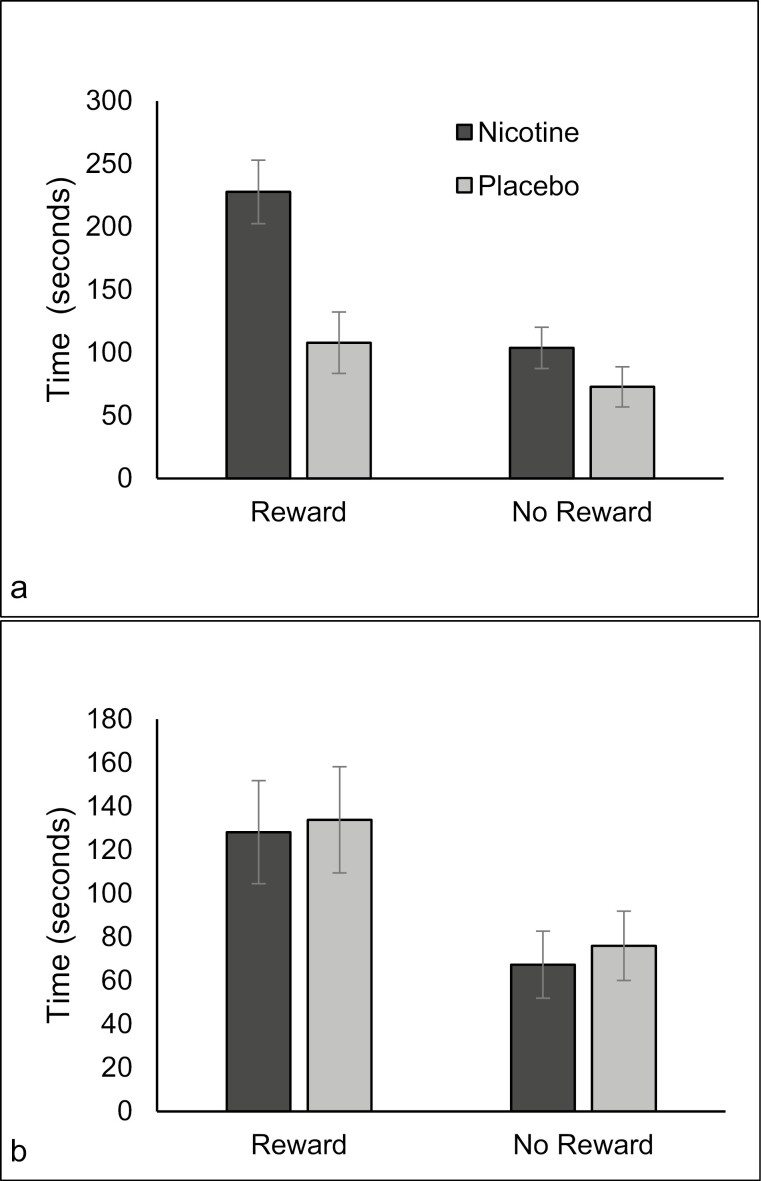
Time spent on task for non-smokers (a) and smokers (b) after nicotine or placebo with and without music rewards.

Time spent on task was higher under the reward condition (M = 149.40, SE = 12.56) than the no reward condition (M = 79.97, SE = 8.18), *F*_(1,57)_ = 45.611, *p* < .001, η_p_^2^ = 0.445. The main effects of spray type (*p* = .071) and smoking status (*p* = .155) were not significant. There were significant two-way interactions between condition and spray type, *F*_(1,57)_ = 4.987, *p* = .029, η_p_^2^ = 0.080, and smoking status and spray type *F*_(1,57)_ = 4.966, *p* = .030, η_p_^2^ = 0.080. These were qualified by a significant three-way interaction between condition, smoking status, and spray type *F*_(1,57)_ = 4.367, *p* < .041, η_p_^2^ = 0.071. The longest time spent on the task was by non-smokers given nicotine under the reward condition (see Figure 3, a and b).

## Discussion

The aim of this study was to explore whether nicotine delivered by oral spray can enhance responding for sensory rewards in both smokers and non-smokers. Participants performed an operant conditioning task where button click responses were required to earn a reinforcer of 30 seconds of their chosen music. Nicotinic enhancement of reward was seen in non-smokers only, who performed the task for longer after nicotine administration whilst their music played. A similar effect has been reported previously in smokers with nicotine delivered by cigarette,^3,4,18,19^ nicotine patch and nasal spray,^21^ and, amongst non-dependent smokers and occasional nicotine users with nicotine delivered by e-cigarette.^21,22^ This study, however, is the first demonstration of this effect in non-smokers via buccal absorption of nicotine. Smokers, however, may require either a higher dose of nicotine to achieve the same effect or a route of administration that more closely replicates the behavioral and sensory aspects of smoking.

All participants earned more reinforcers after receiving nicotine and produced more responses when nicotine was received and music was playing. Non-smokers, but not smokers, produced more responses after receiving nicotine, regardless of whether their music was playing. It is possible that participants found the task itself quite enjoyable and finding reinforcers was rewarding, even in the absence of music. Additionally, nicotine may have enhanced the attention paid to the task goal to find apples (the reinforcer), as nicotine can also improve both smokers and non-smoker’s reaction times, ability to orient attention, and maintain task goals.^30,31^ Number of responses made and reinforcers earned, however, may not be the most informative way to measure the reward value of music when participants are invited to develop their own playlists. One observation made during testing (but not objectively measured) was that response speed varied according to the type of music playing. Participants would click in time to their music. This suggests that time spent on the task was, perhaps, the more reliable indicator of the reward value of the music than the number of responses made or reinforcers earned. Previous studies using self-selected playlists have demonstrated enhanced responding for music rewards after smoking nicotinized cigarettes.^3,18^ E-cigarettes, however, only enhanced responding for video rewards, not music.^20^ It seems unlikely that response speed would vary systematically whilst watching videos, which would mean a high correspondence between the number of responses made and the time spent watching the video. With music, however, slow-tempo music could lower the number of responses made (and in turn, reinforcers earned) in order to listen to, for example, three minutes of their chosen playlist.

One possible explanation for the lack of an effect in smokers in this study is that the amount of nicotine received was not sufficient for people who smoke every day. A 2 mg dose delivered via oral spray is likely to achieve 5 ng/ml of nicotine within 2 minutes of administration.^28^ Levels as high as 30 ng/ml have been reported within 2 minutes of smoking a cigarette^32^ and Patterson et al. suggest that at least 10 ng/ml is required for smokers to experience rewarding effects of nicotine.^33^ Another possibility is that smokers were only required to abstain from smoking for 2 hours prior to participation. Nicotine’s half-life of around 2 hours shows considerable individual variability,^34^ so some smokers may have had pre-existing nicotine levels prior to administration of the spray. Previous studies^3,4,18–21^ have already taken considerable steps to suggest reversal of withdrawal effects is an unlikely explanation for nicotinic enhancement of reward and our data revealed no group differences in nicotine withdrawal symptoms at the start of the session or subjective effects of the sprays.

The finding that nicotine delivered by oral spray enhanced responding for sensory rewards in non-smokers only is generally supportive of a growing literature suggesting that nicotine, and not the sensory aspects of smoking or responses learned via classical conditioning of smoking with pleasurable outcomes, underlie nicotinic enhancement of reward. This may be more pronounced in women, which is also supported by our predominantly female sample of non-smokers.^4^ If so, this would improve our understanding of the anhedonia often reported by smokers during quit attempts that have been linked to the ability to stop smoking longer-term.^35^ The relatively low success rates of nicotine replacement therapy compared to other more popular smoking cessation aids such as e-cigarettes,^36^ however, suggest that smokers require either a larger dose of nicotine than can be obtained via 2 mg nicotine sprays or a form of nicotine administration that more closely replicates the peaks obtained from smoking tobacco. Evidence suggests that experienced e-cigarette users are able to obtain blood nicotine levels from vaping that are similar to those obtained via smoking.^37^ E-cigarettes containing 36 mg/ml have been shown to enhance responding for music and video rewards in smokers,^20^ and 12 mg/ml e-cigarettes increased time spent playing video games in occasional nicotine users.^22^ Given that e-cigarettes are now recognized as successful methods for quitting smoking,^38^ this suggests that nicotine delivered via vaping could provide smokers with some of the benefits obtained from smoking, in terms of improved enjoyment of sensory experiences.

In conclusion, the data reported here confirm that nicotinic enhancement of sensory rewards can be demonstrated in non-smokers and is, therefore, unlikely to be related to nicotine dependence or learned associations between the effects of nicotine and pleasure obtained from smoking. The absence of this effect in smokers suggests that smokers require higher levels of nicotine than those obtained from 2 mg oral sprays to achieve enhancement of reward and, possibly, some replication of the sensory and behavioral aspects of smoking. If, therefore, nicotine replacement, including e-cigarettes, is to become more effective in smoking cessation, smokers would require a dose of nicotine that more closely replicates levels achieved via smoking.

## Supplementary Material

Supplementary material is available at *Nicotine and Tobacco Research* online.

ntae278_suppl_Supplementary_Tables

## Data Availability

Data are currently available via request (email) to the corresponding author. Upon acceptance for publication, data will be made available via the Open Science Framework and/or as required by the journal.
