# Supplementary material for: Acute Effects of Nicotine on Non-Drug-Related Reward in Smokers and Non-Smokers
Source: Nicotine Tob Res. 2025 Feb 5;27(5):815–21. doi: 10.1093/ntr/ntae278 (PMC12012236; doi:10.1093/ntr/ntae278)
Supplement: ntae278_suppl_Supplementary_Tables [file ntae278_suppl_supplementary_tables.docx]

Acute effects of nicotine on non-drug related reward in smokers and non-smokers

Nicola Rycroft, Catherine Kimber, Emke Brazier, Lynne Dawkins

Supplementary analysis to explore changes over time and between groups in desire to smoke and individual items on the MPSS.

A series of 2 (time: time 1 vs time 2) x 2 (smoking status: smoker vs. non-smoker) x 2 (spray type: nicotine vs. placebo) ANOVAs (df 1,57) were conducted.

|  |  | Main effects | | | Two-way interactions | | | Three-way interaction |
| --- | --- | --- | --- | --- | --- | --- | --- | --- |
|  |  | Time | Smoking Status | Spray Type | Time x smoking status | Time x spray type | Smoking Status x spray type | Time x smoking status x spray type |
| Desire to smoke | F | **7.094** | **6.480** | 0.021 | 0.968 | 0.013 | 0.170 | 0.515 |
|  | p | **0.010** | **0.014** | 0.884 | 0.329 | 0.909 | 0.682 | 0.476 |
|  | η_p_^2^ | **0.111** | **0.102** | 0.000 | 0.017 | 0.000 | 0.003 | 0.009 |
| Depressed | F | **12.060** | 0.081 | 0.362 | 0.762 | 0.001 | 0.084 | 0.688 |
|  | p | **0.001** | 0.788 | 0.550 | 0.386 | 0.970 | 0.772 | 0.410 |
|  | η_p_^2^ | **0.175** | 0.001 | 0.772 | 0.013 | 0.000 | 0.001 | 0.012 |
| Irritable | F | 3.523 | 1.253 | 0.304 | 1.457 | 0.113 | 0.037 | 0.008 |
|  | p | 0.066 | 0.268 | 0.584 | 0.232 | 0.738 | 0.847 | 0.929 |
|  | η_p_^2^ | 0.058 | 0.022 | 0.847 | 0.025 | 0.002 | 0.001 | 0.000 |
| Anxious | F | **10.705** | 2.003 | 0.124 | 1.640 | 0.293 | 0.729 | 0.731 |
|  | p | **0.002** | 0.162 | 0.726 | 0.206 | 0.591 | 0.397 | 0.396 |
|  | η_p_^2^ | **0.158** | 0.034 | 0.397 | 0.028 | 0.005 | 0.013 | 0.013 |
| Restless | F | **6.306** | 3.043 | 2.382 | 3.006 | 1.688 | 1.047 | 0.746 |
|  | p | **0.015** | 0.086 | 0.128 | 0.088 | 0.199 | 0.311 | 0.391 |
|  | η_p_^2^ | **0.100** | 0.051 | 0.040 | 0.050 | 0.029 | 0.018 | 0.013 |
| Hungry | F | 0.280 | 0.237 | 0.090 | 1.535 | 0.052 | 0.610 | 0.121 |
|  | p | 0.598 | 0.628 | 0.765 | 0.220 | 0.821 | 0.438 | 0.729 |
|  | η_p_^2^ | 0.005 | 0.004 | 0.002 | 0.026 | 0.001 | 0.011 | 0.002 |
| Inability to concentrate | F | 2.910 | 0.495 | 0.457 | 0.002 | 0.838 | 0.321 | 0.378 |
|  | p | 0.093 | 0.485 | 0.502 | 0.969 | 0.364 | 0.573 | 0.541 |
|  | η_p_^2^ | 0.049 | 0.009 | 0.008 | 0.000 | 0.014 | 0.006 | 0.007 |

Supplementary Table 1. F, p and η_p_^2^ values for 2x2x2 ANOVAs on craving for cigarettes and individual items on the MPSS.

The significant main effects of time showed an increase in desire to smoke, depression, anxiety and restlessness from the start to the end of the testing session, see Supplementary Table 2. The main effect of smoking status revealed higher levels of desire to smoke amongst smokers (mean = 4.59, SE = 0.74) than non-smokers (mean = 1.97, SD = 0.72).

|  | Time 1 | Time 2 |
| --- | --- | --- |
| Desire to smoke | 1.96 (0.14) | 4.60 (1.01) |
| Depression | 1.02 (0.10) | 1.99 (0.30) |
| Anxiety | 1.35 (0.12) | 8.30 (2.14) |
| Restlessness | 1.41 (0.13) | 5.37 (1.62) |

Supplementary Table 2. Mean (SE) for the main effects of time in craving and MPSS items.

Due to the lack of interactions with smoking status or spray type, these main effects appear to show changes in mood from the start to the end of the testing session that occurred regardless of smoking status and which spray type was administered.
